# Medical Education in the Time of the Great War

**Published:** 1917-09-08

**Authors:** 


### The Hospital

The Workers' Newspaper of Administrative Medicine and Institutional Life, Administration, National Insurance and Health.

No. 1631, Vol. LXII. SATURDAY, SEPTEMBER 8, 1917. PRICE ONE PENNY

### MEDICAL EDUCATION

### IN THE TIME OF THE GREAT WAR.

Like many other activities which have an abiding value, medical education may not be stayed even by the stress and shock of war. Boys are growing into manhood whatever the international situation, and to adventurous youth there comes inevitably the hour of choice and of decision. In our modern day the summons to war claims many who would otherwise be selecting or pursuing a medical career, but circumstances of various kinds still leave to some the opportunity of a peaceful calling. Fortunate it is that disciples are possible, for the years will surely need them. Even the most gloomy of pessimists admits that the war cannot go on for ever, and that foresight must take an account of the approach of peace. Whatever else be true of the re-establishment of the normal order, it is certain that medical services will find even larger opportunities than they have enjoyed in the past. Death and disablement suffered on the battlefield have diminished the numbers of the active workers, and many potential students of medicine have followed the call to arms. Hence both in the present, and for several succeeding years, the medical profession will see admitted to its ranks but a scanty band of recruits. The life of the nation, however, is going to need not less but more expert medical service. There can thus be no fear in the minds of the new candidates that opportunity will fail them. The exact opposite is the case. Opportunity is going to be multiplied and dignified, and the resolute and strong of heart may be welcomed to the enterprise.

The medical student of to-day comes at a time when many new and quickening influences are stimulating the atmosphere of professional life. The older traditions remain, and their value and force are in no degree lessened. But the hour is rich with fresh thoughts and wide prospects. Never more than now has the contribution offered by medicine to the national welfare been more conspicuous or more secure of recognition. A hundred battlefields proclaim the cool and reasoned heroism of its followers, and the health of our armies and our camps displays how effectively science has been linked to practical ends. The ranks of our fighting forces are full and vigorous because medicine protects them from a thousand secret and insidious foes. Bullets claim their victims but bacilli are held in secure subjection. The story of our campaigns, in short, is a proclamation of the triumphs of medical, surgical, and sanitary achievement, though administrative efficiency has a less unbroken record. To these great results, and to the lustre which accompanies them, our new students enter as to an inheritance, and there is stimulus in the thought that they may prove themselves worthy of so fair a gift. The added motive to be put to them is that what is now evident to all eyes is the issue and result of prolonged, sustained, and directed effort. The stage has been set up in the sight of all the world, but the message to the student is of the skilful and unwearied preparations which have made so great a performance possible. Great ends mean wise beginnings and continued and consistent adhesion to daily duty. Not to him who merely waits, but to him who knows how to wait, in self-discipline and apt effort, does fate give the opportunity of large service and telling triumph. This message just now is spoken to the student with new force and in the midst of events which arouse every mind. What is seen as an active success to-day was made possible by quiet and persevering labours in the hospitals and laboratories and lecture-rooms of yesterday, and similarly the fate of to-morrow is being shaped in the present hour. It is in presence of this old-world truth emphasised by a stirring and dramatic setting that our new students enter on their career.

What is the word which a contemplation of the future has to speak to them? Though perhaps less vivid than that of the history of the moment, it is not less encouraging. The possession of health and the importance of bodily fitness have acquired new values in the national judgment. Large bodies of men who are going to provide the public opinion of the immediate future have learned in the school of practical experience the sense of well-being and personal capacity bestowed by physical discipline and the open-air life. The national catastrophe implied in the loss of infant life and in the failure to prevent preventable disease has burned itself into the national conscience. Health education is seen to be of the first importance, not merely to the individual but to the community, and this both for the triumphs of war and also for the economies of peace. Towards all these ends there is a resolute and increasing movement, and in every one of them medicine must play a great and commanding part. It is to fit themselves for this part that the students of to-day are summoned. Never have medical activities been wholly separated from public interests, but the future is going to link the two in a closer and more conspicuous union, and the new generation of practitioners is called to equip itself for this larger development. So long as disease and mischance attend on human fate the individual victim will claim the relief which medical capacity can afford. For this function here and now, not less than in former days, the student must prepare himself, and the ambition is a worthy one. But in an increasing measure he is going to be called upon to prove himself able to educate the public in the methods of health preservation and of bodily culture. The task will lead him into relations with social enterprises of various orders, and he will have to inform his mind with a knowledge of circumstances and conditions which influence the life and vigour of great bodies of our people. If wisdom is to attend on our counsels the nation must have the educated direction and guidance of a competent, informed, and sympathetic medical profession, and on such a prospect our newcomers may surely look as on a future equal to the most eager of ambitions.

